# Nutraceuticals in Viral Infections: An Overview of the Immunomodulating Properties

**DOI:** 10.3390/nu13072410

**Published:** 2021-07-14

**Authors:** Giorgio Costagliola, Giulia Nuzzi, Erika Spada, Pasquale Comberiati, Elvira Verduci, Diego G. Peroni

**Affiliations:** 1 Department of Clinical and Experimental Medicine, Division of Pediatrics, University of Pisa, Via Roma 57, 56126 Pisa, Italy; giorgio.costagliola@hotmail.com (G.C.); giulianuzzi92@gmail.com (G.N.); erika.spada@virgilio.it (E.S.); pasquale.comberiati@gmail.com (P.C.); 2 Department of Clinical Immunology and Allergology, I.M. Sechenov First Moscow State Medical University, 119435 Moscow, Russia; 3 Department of Pediatrics, San Paolo Hospital, 20142 Milan, Italy; elvira.verduci@unimi.it; 4 Department of Health Science, University of Milan, 20142 Milan, Italy

**Keywords:** polyphenols, resveratrol, vitamin A, vitamin D, zinc, lactoferrin, magnesium, selenium, coenzyme Q

## Abstract

Nutraceuticals, including vitamin D, vitamin A, zinc, lactoferrin, polyphenols coenzyme Q, magnesium, and selenium, are implicated in the modulation of the complex molecular pathways involved in the immune response against viral pathogens. A common element of the activity of nutraceuticals is their ability to enhance the innate immune response against pathogens by acting on the major cellular subsets and inducing the release of pro-inflammatory cytokines and antimicrobial peptides. In some cases, this action is accompanied by a direct antimicrobial effect, as evidenced in the specific case of lactoferrin. Furthermore, nutraceuticals act through complex molecular mechanisms to minimize the damage caused by the activation of the immune system against pathogens, reducing the oxidative damage, influencing the antigen presentation, enhancing the differentiation and proliferation of regulatory T cells, driving the differentiation of lymphocyte subsets, and modulating the production of pro-inflammatory cytokines. In this paper, we review the main molecular mechanisms responsible for the immunomodulatory function of nutraceuticals, focusing on the most relevant aspects for the prevention and treatment of viral infections.

## 1. Introduction

**Figure 1:**
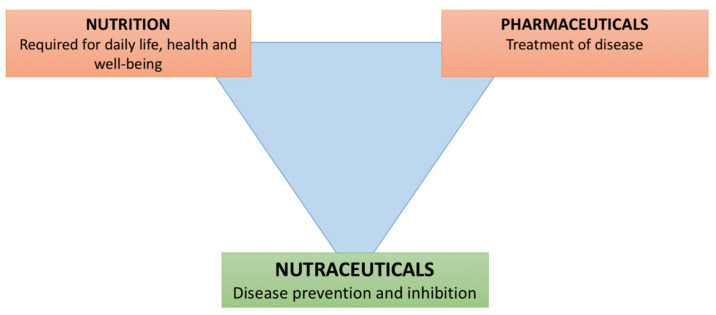
Role of nutraceuticals in health and disease.

Nutraceuticals are products derived from food that play a role in maintaining well-being, enhancing health, modulating immunity, and thereby preventing as well as treating specific diseases (Figure 1) [1]. Nutraceuticals have complex interactions with the immune system, with the potentiality to improve the response against pathogens but also to modulate the activation of immunity itself [2]. Given their immunomodulatory activities, for years they have been the subject of studies not only to clarify their complex biological mechanisms, but also to assess their possible applications in clinical practice, for the maintenance of good general health and the prevention of pathological conditions including infections, chronic inflammatory or autoimmune diseases, and allergic diseases [3]. Concerning viral infections, the optimal functioning of the innate and adaptive immune response is essential in the defense against pathogens and allows the clearance of the infectious agent and the generation of an immunological memory, which prevents reinfection by the same pathogen. However, when the innate or adaptive immune response is activated in an uncontrolled way, the immune system itself could be responsible for the development of tissue damage, thus representing a “double-faced weapon”. Therefore, there is increasing interest in strategies to potentially modulate the activation of the immune response against viral infections, to reduce immune- and inflammatory-induced organ damage while maintaining (and potentially enhancing) the capability to prevent infection or adequately eliminate the infectious agent. In this paper, we review the main molecular mechanisms responsible for the immunomodulatory function of nutraceuticals, with a particular focus on vitamin D, vitamin A, lactoferrin, zinc, polyphenols, coenzyme Q, magnesium, and selenium, in order to update clinicians on the benefit of using nutraceuticals for the management and the prevention of allergic diseases and viral infections.

## 2. Vitamin D

Vitamin D (VD) is a fat-soluble hormone mainly synthesized in the skin during exposure to sunlight (UVB radiation) through the conversion of 7-dehydrocholesterol to biologically inactive vitamin D3. This undergoes hydroxylation in the liver by 25-hydroxylase to become 25(OH)D, the major circulating form of VD in humans. In the kidney, 25(OH)D is further converted to the active metabolite 1,25(OH)D by the 1-α-hydroxylase [4]. Vitamin D 1,25-OH acts on target cells via the VD receptor (VDR), which binds specific sequences, called “VD responsive elements”, through which it acts as an activator or repressor of transcription of several genes. Although VD plays a fundamental role in phospho-calcium homeostasis, recent studies have shown that it has numerous extra-skeletal actions, including important immunostimulant and immunomodulatory properties. These observations suggest a key role for VD in the modulation of immune function, above all during pediatric age, as high levels of VD are important to prevent viral respiratory infections. A relationship between VD status and the incidence or the severity of respiratory infections in children has been found in many observational studies, and a recent review seems to support the protective role of VD against tuberculosis, otitis media, pharyngotonsillitis, bronchiolitis, and viral wheezing [5,6,7]. Even during the recent COVID-19 pandemic, VD and its metabolites were found to be useful both in inducing the antiviral response and in negatively regulating the renin-angiotensin-aldosterone system, including the expression of ACE2, the receptor for SARS-CoV2 [8,9]. The influence of VD status on the clinical outcome of patients with COVID-19 is still undefined, as studies have shown conflicting results [10,11,12]. However, there is increasing interest in the potential influence of several age-related factors (function and maturation of innate and adaptive immunity, ACE2 expression, comorbidities) and the nutritional status on the clinical course of SARS-CoV-2 infection [13,14]. Although systemic levels of VD are regulated on phospho-calcium metabolism, tissue production of VD by cells expressing 1α-hydroxylase is regulated by calcium-independent mechanisms and can significantly influence the immune response through autocrine and paracrine mechanisms [4,15].

### 2.1. Antimicrobial Activity

The action of VD on the immune system is extremely complex, as VDR is expressed by many cells involved in the inflammatory response and the innate and adaptive immune response. Indeed, VD modulates cytokine secretion, through its action on the VDR receptor, and controls the function of lymphocyte subpopulations, both directly and by influencing the function of antigen-presenting cells (APCs), which are responsible for the expression of co-stimulatory molecules that are essential for the activation of the immune response [16]. Cells of innate immunity and epithelial cells, particularly in the airways, can synthesize vitamin D 1,25-OH locally through the expression of 1α-hydroxylase. The expression of this enzyme is stimulated by various mechanisms, including the action of interferon (IFN-γ), the activation of toll-like receptors (TLRs) involved in the innate immune response, and the cellular pathways they control (JAK/STAT pathway, NF-kB pathway), and is, therefore, a phenomenon that amplifies the immune response itself [16,17]. In addition, VD increases the chemotaxis of cells of innate immunity, their microbicidal activity, autophagy, and the production of defensins and cathelicidins, soluble mediators with an antimicrobial function [18]. VD also contributes to the immune response by causing an enhancement of epithelial barrier function in both the skin and the lung, stimulating the maturation of type II pneumocytes, promoting surfactant production, and increasing the innate immune response in airways. In the gut, VD induces the expression of occludins and claudins, thereby increasing the integrity of the mucosal barrier. In addition, intestinal mucosal homeostasis is also influenced by modulation of the local microbiome, which in turn is essential in the development of immunological tolerance and is linked to the systemic immune system via a complex system of interactions known as the “gut-immune system axis”. This action ultimately results in a protective effect against gastrointestinal infections and modulation of the secretion of pro-inflammatory cytokines [19] (Table 1).

### 2.2. Immunomodulation

VD prevents excessive activation of the inflammatory and immune response by reducing the excessive release of interleukin-1β (IL-1β) and IL-6, as demonstrated in patients with sepsis. VD contributes to maintaining adequate plasmatic levels of dendritic cells (DCs) and promotes a “tolerogenic” phenotype of dendritic cells (DCs), which is featured by reduced production of IL-12 and increased synthesis of IL-10 [17,19]. Additionally, VD reduces the activation of T-helper-1 (Th1) and Th17 cells, resulting in reduced production of pro-inflammatory cytokines (IFN-γ, IL-12, and IL-17) and the consequent tissue damage [18,20]. Finally, VD promotes the differentiation and proliferation of regulatory T cells (Tregs) and Th2 cells in both healthy individuals and patients affected by inflammatory disorders [21,22], influences lymphocyte homing (and, therefore, the activation of adaptive immunity), and causes a reduction of memory B cell differentiation and immunoglobulin production [14,23,24]. The molecular mechanisms responsible for these effects are not completely elucidated. The influence of VD on T CD4+ cells is extremely complex and involves the stimulation of the FOXP3 and GATA3 transcription factors (which drive Treg and Th2 proliferation, respectively) and the inhibition of IL17A gene expression [18]. On the other hand, the effects on B cells and immunoglobulins are mostly mediated by the negative regulation of Nf-KB and the reduction of costimulatory signals [25], while the basis of the action on DCs is far from being fully understood.

Concerning the threshold for VD supplementation, several scientific societies have established that 25(OH)D levels higher than 20 ng/mL are sufficient to ensure optimal bone health, while 25(OH)D levels higher than 30 ng/mL are needed to favor VD extra-skeletal actions [26,27]. Notably, the use of VD supplementation in the prevention and treatment of several immune-mediated disorders (systemic lupus erythematosus (SLE), multiple sclerosis, inflammatory bowel diseases) has shown promising results [18]. Moreover, the influence on the immune response (particularly on thymic output and DCs) has been demonstrated also in patients with immunodeficiency affecting T-cell function, which represents a population carrying a higher risk of severe viral infections [28]. Therefore, the identification of high-risk patients could lead to an optimization of VD supplementation strategies.

## 3. Vitamin A

The term vitamin A (VA) refers to a large group of fat-soluble elements, including retinol, retinoids, and carotenoids. The active metabolite retinoic acid is of particular clinical interest, as it is involved in numerous functions, such as regulation of cell differentiation, control of oxidative stress, and modulation of the immune response. Retinoic acid is synthesized through a series of enzymatic reactions and binds to its nuclear receptor (retinoic acid receptor: RAR), which interacts with specific sequences, termed “retinol-responsive elements” at the promoter level of target genes, influencing their transcription [29]. The suggested daily requirement of VA, expressed in micrograms of retinol (1 mcg retinol = 6 mcg beta-carotene = 12 mcg other provitamin carotenoids), is 450 mcg in infants, 300–500 mcg in school-age children, and 600–700 mcg in adolescence. VA deficiency is defined by plasma concentrations of less than 20 mcg/dL. Currently, the World Health Organization (WHO) recommends supplementation with 100,000 IU of vitamin A between 6 and 12 months of age and 200,000 IU from 12 months to 5 years of age, taken every six months orally [30]. VA supplementation has also been shown to be effective in the prevention and treatment of viral gastrointestinal infections in terms of reduced severity and days of illness; zinc and VA supplementation has been associated with faster resolution of diarrheal symptoms in developing countries [31,32].

### 3.1. Antimicrobial Activity

The action of retinoic acid on the immune system is of particular importance with regard to gastro-intestinal mucosal immunity, where it can influence the proliferation, activity, and migration of APCs and cells of adaptive immunity [33]. Similar to VD, VA can modulate the composition of the gut microbiome and directly stimulate the synthesis of proteins involved in maintaining the integrity of the gastrointestinal barrier [34]. Indeed, VA deficiency is associated with an increased burden of infectious disease [35]. The anti-infective and immunomodulatory actions of retinoic acid partly depend on its synthesis by DCs. In particular, DCs located in the mesenteric lymph nodes and Peyer’s patches, and those located in the skin and lungs, express the enzyme retinal dehydrogenase (RALDH) and can synthesize retinoic acid both constitutively and in response to infectious stimuli (bacteria, viruses, parasites) [29,34]. Through the interaction with retinoid X receptors (RXRs), retinoic acid is involved in modulating the function of DCs themselves and their ability to present antigens as well as stimulating the differentiation of specific dendritic cells with a tropism for the intestinal mucosa. Indeed, it promotes their maturation, activation, antigen-processing, and antigen-presenting capacity and may also influence the maturation and tissue tropism of T and B lymphocytes, also enhancing the proliferation of T lymphocytes and increasing their cytotoxic activity [34,36]. The action on T cells partly depends on the enhancement of IL-2 secretion and signaling, IL-22 production, and the expression of gut-homing receptors [24]. Finally, it stimulates isotypic switch and IgA production, thus significantly improving mucosal immunity [37]. This latter effect, which requires adequate mucosal concentrations of IL-5 and IL-6, is mediated by the stimulation of the inducible nitric oxide synthase (iNOS) enzyme and by the modulation of the gut-associated lymphoid tissue (GALT) DCs activity [24,38]. Accordingly, in experimental models of VD deficiency, a reduced mucosal IgA concentration was observed [24,39].

### 3.2. Immunomodulation

**Figure 2:**
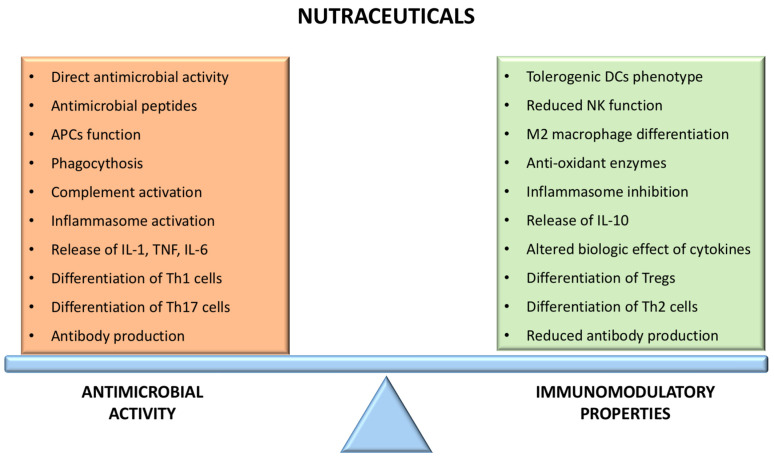
Action of nutraceuticals on the immune balance.

VA is not only involved in immunomodulation during the infectious response but also seems to play an important role in modulating the inflammatory and immune response (Figure 2). Indeed, it could influence the Th1-Th2 balance, favoring the Th2 response [40]. This effect mostly depends on the influence on transcription factors, including the stimulation of GATA3, STAT6, and IL4 genes (implicated in Th2 differentiation) and the inhibition of Tbet, which is involved in Th1 differentiation [41]. Additionally, VA induces Treg differentiation, both directly (activating FOXP3) and through its action on DCs, and promotes Treg tropism for the intestinal mucosa by stimulating the expression of gut-specific receptors (i.e., CCR9 and α4β7 integrin) [42]. Interestingly, the modulation of different transcription factors by retinoic acid participates in the regulation of the Treg-Th17 balance, as the differentiation of these subpopulations is mutually regulated. Experimental studies demonstrated that low concentrations of retinoic acid could promote Th17 differentiation and inhibit Tregs, while higher concentrations mediate the opposite effects, finally contributing to mucosal immune tolerance [39]. Interestingly, it has recently been shown that retinol deficiency in transplant patients is associated with a higher incidence of Graft Versus Host Disease (GVDH), especially intestinal, through reduced gastrointestinal permeability, higher rate of mucosal injury, and reduced lymphocyte homing to the gut [43].

## 4. Zinc

Zinc has a key role in numerous signaling pathways and cellular processes involved in the activation and modulation of the immune response to viral infections [44]. Studies on the use of zinc in the prevention and treatment of respiratory and gastrointestinal viral infections have shown conflicting results. Indeed, while many studies demonstrated that zinc could reduce the incidence and severity of upper respiratory infections, other authors found no effect, and zinc seems not to influence the prevention and clinical course of lower respiratory infection [3,9].

### 4.1. Antimicrobial Activity

At a molecular level, zinc can act as a signaling molecule, to reversibly bind the regulatory subunits of proteins implicated in immune pathways and to regulate the composition and permeability of the cell membrane [45,46]. Moreover, it is essential in maintaining the structural and functional integrity of different enzymes (kinases, phosphatases, caspases, and others) involved in the immune response [45]. Interestingly, the acute (zinc flux, zinc waves) and chronic (homeostatic zinc signals) variations in cellular zinc concentration can activate different molecular pathways, and this could partly explain the promoting effect on immune activation during acute antigenic stimulation (viral infection) and the inhibition of the immune response following the chronic stimulation of the immune system. The anti-infectious properties of zinc depend on the direct interaction with viral replication and on the stimulation of antiviral immunity. In vitro studies have demonstrated that zinc can inhibit viral replication of different pathogens of the upper and lower airways, including influenza A virus and respiratory syncytial virus (RSV), with a mechanism that involves the interference with DNA and RNA polymerase activity [9,47]. The enhancement of the innate immune response against pathogens strongly contributes to zinc’s antiviral properties. In vitro, the binding of TLR4 with the lipopolysaccharide (LPS) is followed by an acute intracellular zinc flux, which promotes the activation of NF-kB, central in the production of acute-phase proteins and pro-inflammatory cytokines [48]. Moreover, zinc stimulates the production of IFN and other antiviral proteins as well as the proliferation of cells involved in the innate (macrophages, neutrophils, natural killer (NK) cells) and adaptive immune response and enhances NK cell lytic activity and function [49]. Concerning adaptive immunity, zinc has a key role in the differentiation and proliferation of T cells, being a cofactor for the thymocyte-derived hormone thymuline [44,50]. Although the proliferation of B cells seems not to be influenced by zinc [51], it promotes antibody production and influences isotypic switch through the influence on specific transcription factors and cytokine signaling (i.e., IL-4-mediated phosphorylation of STAT6 in the class switch favoring IgE production) [45,52]. Accordingly, patients with zinc deficiency show reduced adaptive immune response and higher susceptibility to the development of severe infections compared to the general population [49]. However, recent studies suggest that zinc deficiency is also more common in patients with autoimmune diseases, thus underlying its role in modulating the immune response [53].

### 4.2. Immunomodulation

Persistent antigenic stimulation (i.e., in patients with chronic viral infections or autoimmune diseases) produces chronically elevated cellular levels of zinc. This long-term elevation of zinc levels causes NF-kB inhibition, with consequently reduced release of pro-inflammatory cytokines (IL-1β, tumor necrosis factor-α (TNF-α)), lipid peroxidation, and release of reactive oxygen species (ROS) [46,49,50,54]. The mechanism underlying the inhibition of Nf-KB seems to be mostly dependent on the effect of zinc on the anti-inflammatory zinc-finger protein A20 and the peroxisome proliferator-activated receptor-α (PPAR-α) [46]. The immunomodulatory role of zinc depends also on its effect on adaptive immunity. Indeed, zinc is implicated in the enhancement of TGF-β-induced signaling and in the activation and stabilization of FOXP3, thus finally driving the differentiation of CD4+ lymphocytes into Tregs. The influence of zinc on lymphocyte subpopulation is also confirmed by the reduced Th1 response, with a Th1-Th2 imbalance observed in patients with zinc deficiency, although the molecular mechanism is not well defined [51]. Finally, zinc influences the intracellular cascades that follow the activation of the T-cell receptor (TCR), B-cell receptor (BCR), and cytokine receptors. It regulates the expression of NFAT transcription factor, which promotes IL-2 production in activated lymphocytes and inhibits calcineurin (implicated in NFAT activation) [45,55], but also enhances the phosphoinositide 3-kinase (PI3K)-induced signaling, activated after the binding of TCR and BCR with their ligands. Therefore, zinc interferes with a wide range of activating and inhibitory molecular pathways in the complex network that follows lymphocyte activation [45,56]. Concerning cytokines, in vitro and experimental studies have evidenced that zinc deficiency causes an enhanced biological effect of IL-6 and IL-2 [54], which are implicated in the maintenance of the inflammatory response and lymphocyte proliferative response. On the other hand, zinc deficiency has the opposite effect on IL-4 signaling, thus resulting in an imbalanced immune function [52].

## 5. Lactoferrin

Lactoferrin is a glycoprotein physiologically present in breast milk, with higher concentrations in colostrum, and represents a key factor in the newborn innate immune system [57,58], with its supplementation in pre-terms being associated with a reduced incidence of late-onset sepsis [59]. The antibacterial properties of lactoferrin are well known and derive from its capability to bind and sequestrate iron (bacteriostatic effect), from the direct bactericidal effect (particularly in Gram-negative LPS-producer bacteria), and from the capability to activate the complement cascade [60,61,62]. In addition, the antiviral and immunomodulatory properties of lactoferrin are of great interest and have been the subject of a wide number of clinical and preclinical studies. Although preclinical studies have helped in clarifying the main molecular mechanisms, the clinical applications in the field of viral infections are still to be completely defined. However, the most recent clinical studies investigating the effect of lactoferrin supplementation on the prevention and treatment of viral infections showed promising results, as different authors evidenced a reduction of the frequency and severity of viral gastroenteritis in infants and a reduced incidence of cold in adults [57,63,64].

### 5.1. Antimicrobial Activity

The antiviral effects of lactoferrin depend on the inhibition of different phases of the infectious cycle. Indeed, it can interfere with the binding of viral pathogens with heparin sulfate glycosaminoglycan molecules on the cellular surface, thus blocking the interaction between the virus and its receptor and its entry into the host cell [57,60]. In vitro studies performed on different pathogens (influenza A virus, parainfluenza virus, RSV, and others) evidenced that lactoferrin inhibits viral replication, with an effect that is more pronounced when adequate cellular zinc levels are present [9,57]. Additionally, lactoferrin has complex immunostimulating properties that promote viral clearance, enhancing both innate and adaptive responses [65]. The effect of lactoferrin on the different cellular targets is mediated by its interaction with cellular receptors (surface peptidoglycans and glycosaminoglycans, TLRs, and others), which promote its internalization. Following this, lactoferrin can act through the modulation of signaling pathways (interacting with kinases and phosphatases) and transcription factors [62]. Concerning the innate response, lactoferrin acts as a chemotactic factor for leukocytes, enhancing cellular activity, cytokine release (particularly IFN), and function of NK cells (thus promoting phagocytosis), neutrophils, and macrophages, and participates in reinforcing and maintaining the integrity of the gut mucosal barrier [61,66]. Different studies have shown that lactoferrin accelerates the maturation of enterocytes (mainly through the activation of MAP kinase (MAPK)-dependent molecular pathways), thus reducing the permeability of the mucosal barrier and the systemic spreading of infectious agents, and promotes the development of the gut microbiome [65,67]. This latter effect is of particular relevance in newborns, since the microbiome prevents gut colonization by pathogens [66] and has a direct local and systemic immunomodulatory role through the stimulation of the release of IL-10 [68] and the downregulation of Th1- and Th17-dependent cytokines [69]. The action on innate immunity is also partly responsible for the effects of lactoferrin on the adaptive immune response. Indeed, lactoferrin stimulates the maturation and activation of DCs and macrophages and enhances their antigen-presenting function and secretion of cytokines (such as TNF-α, IL-8, and IL-12), finally promoting a Th1-mediated immune response [70]. Additionally, lactoferrin directly influences lymphocyte maturation and proliferation, as different experimental studies showed that lactoferrin enhances the maturation of T helper lymphocytes and B lymphocytes and their antigen-presentation function as well as the production of immunoglobulin, both at the mucosal level and systemically [65,71,72]. Activation of MAPK pathways contributes to the promoting effect of lactoferrin on T cell maturation, while the enhanced release of IL-12 is important in driving Th1 differentiation [65,73].

### 5.2. Immunomodulation

The recently described immunomodulatory activity of lactoferrin mostly depends on its influence on the function of innate immune cells and mediators. Lactoferrin can cause a reduced inflammatory response by pathogen-infected cells and reduced activation of antigen-presenting cells (particularly DCs) [74] in case of exuberant inflammation. Additionally, it can reduce the release of a wide range of pro-inflammatory cytokines, including TNF-α and IL-6 [61,75], the production of chemotactic factors, and the expression of adhesion molecules, such as ICAM-1 and E-selectin [65,76]. At the molecular level, these effects partially depend on a direct interference with the TLR4-LPS interaction, through the binding of circulating LPS and the interaction with TLR4 [70,77]. Moreover, lactoferrin can bind to TLR2, TLR9, and other danger signal receptors, thus influencing the intracellular pathways of the inflammatory response [78]. The inhibition of lipid peroxidation and the induction of enzymes with antioxidant properties (i.e., superoxide dismutase) contribute to the reduction of the ROS-induced damage [79,80], which has a relevant role in viral infections. The effects of lactoferrin on the adaptive immune system are multiple, as the molecule can promote both the stimulation and the inhibition of lymphocyte proliferation and differentiation, with mechanisms that are not completely defined [76]. Concerning this, in contrast with the previously described effects on the promotion of adaptive response, both in vitro and experimental studies have evidenced that lactoferrin can reduce the activity of the TLR-NF-kB axis [77,79]. This mechanism partially contributes to the effect of lactoferrin in reducing T cell activation (particularly the Th1 response) and secretion of pro-inflammatory cytokines [70], thus minimizing the tissue damage that follows the activation of the immune and inflammatory response against pathogens.

## 6. Polyphenols

The polyphenol family includes a wide number of molecules, classified into flavonoids and non-flavonoids, of which resveratrol is the most studied for its clinical applications. Single molecules of the polyphenol family can show different interactions with the cells and mediators of the immune system, and both immunostimulating and immunomodulating effects are observed [81,82]. Clinical studies on viral infections are still limited, but evidence suggests a promising role for resveratrol in the prevention and mitigation of respiratory infections [83].

### 6.1. Antiviral and Immunomodulating Properties of Resveratrol

The molecular actions of resveratrol contribute to favoring the clearance of viral pathogens and the reduction of immune- and inflammation-mediated damage. Concerning the antiviral properties, in vitro studies have demonstrated that resveratrol can inhibit the replication of RSV, SARS-CoV2, and other viral pathogens, and interferes with different phases of the viral replication cycle [84,85].

The immunomodulating properties of resveratrol are mostly mediated by its interaction with SIRT1, a protein implicated in numerous signaling and transcription pathways, modulation of immune cell proliferation and activation, inhibition of cyclo-oxygenase 2 (COX-2) enzyme, and regulation of oxidative damage [86]. The antioxidant role of resveratrol has been extensively studied and depends on its ability to influence mitochondrial functioning [87]. Indeed, resveratrol causes transcriptional modifications that carry an enhanced expression of superoxide dismutase, catalase, and glutathione peroxidase, thus reducing cellular oxidative stress [87,88,89]. The stimulation of SIRT1 is also involved in the downstream of NF-kB-dependent molecular pathways in cells of the innate and adaptive immune system [86]. This effect, which partly depends on the interaction with the Nf-KB subunit p65 and on the degradation of the inhibitor protein-κBα (IkBα), is responsible for a reduced secretion of TNF-α, IL-1β, IL-6, and other inflammatory mediators by innate immune cells [86]. Experimental studies have shown that resveratrol can reduce the surface expression of TLRs (particularly TLR4) by cells of the innate immune system and lead to a downregulation of TLR-mediated response (i.e., MAPK activity) [86,90]. This effect is particularly relevant for patients with chronic viral infections and immune-mediated diseases, such as SLE, in which TLR activation plays an important pathogenic role [91]. However, the effects of resveratrol on the release of cytokines are not of unequivocal interpretation, as in vivo studies have evidenced that supplementation with resveratrol reduces the serum levels of macrophage-derived cytokines, while those produced by lymphocytes were found to be augmented [92]. Concerning this, it is reasonable to hypothesize that resveratrol could reduce the chronic inflammatory burden evidenced in several immune-mediated diseases and chronic infections while enhancing the systemic adaptive response to viral pathogens. The immunomodulatory effects on adaptive immunity include the reduced proliferation and differentiation of B cells and Th17 cells, reduced antibody production, enhanced production of IL-10, and interference with the activation of self-reactive T cells, while the influence on the absolute number of T-regs is controversial [86,93,94]. The effects on T cells are mediated by Sirt1, which blocks the translocation of c-Jun (central in the progression of cell cycle and inhibition of apoptosis) to the nucleus [95]. On the other hand, the modulation of humoral immunity involves the upregulation of the inhibitory molecule FcγRIIB on B cells, which results in decreased antibody production [86].

### 6.2. Antiviral and Immunomodulating Properties of Other Polyphenols

Quercetin is a flavonoid with marked anti-inflammatory properties. In vitro studies have shown that quercetin can reduce NF-kB activation (blocking the nuclear translocation of p50 and p65 subunits) [82], the release of pro-inflammatory cytokines after LPS stimulation, and the production of ROS by directly inhibiting COX-2 and lipid peroxidation [96]. In addition, quercetin influences the expression of adhesion molecules (i.e., VCAM-1) and the release of metalloproteinases, thus contributing to the reduction of inflammatory tissue damage [96]. This anti-inflammatory effect has also been confirmed by experimental studies [97], while clinical data are still conflicting [96]. On the other hand, quercetin promotes Th1 response while reducing the secretion of Th2-produced cytokines [96,98], thus contributing to the host defense against viral infections. The actions of quercetin on lymphocyte function are complex, and the underlying molecular mechanisms are still undefined. However, the modulation of PI3K, protein kinase C (PKC), and JAK-STAT pathways plays a significant role [96,99,100]. Similar effects are evidenced in studies on the non-flavonoid curcumin. Indeed, this molecule can reduce the secretion of pro-inflammatory cytokines, the production of ROS and nitric oxide, and the expression of endothelial adhesion molecules and TLR-dependent signaling at different levels (downregulation of TLR expression, inhibition of TLR-activated kinases) [101,102]. Furthermore, in vitro studies show that curcumin impairs the antigen-presenting function of DCs [103], thus preventing the activation of adaptive immunity. Finally, curcumin modulates the activation of a wide number of transcription factors implicated in the immune response, including STAT3 and NF-kB, by blocking their translocation to the nucleus or the interaction with DNA [102], finally leading to complex, and not completely understood, effects on immunity [101,104].

## 7. Other Nutraceuticals with Immunomodulatory Properties

Among the other nutraceuticals with immunomodulatory activity, the roles of magnesium, selenium, and coenzyme Q are of particular interest in the field of viral infections.

Magnesium is an essential cofactor for different reactions implicated in the response against viral infection and immune regulation, having a role in T cell development, antibody production, and cytokine release and signaling [105,106]. This function depends on multiple mechanisms, including the inhibition of NF-kB signaling, the regulation of apoptosis, and DNA replication and repair [2,107]. At the molecular level, magnesium acts by modulating the function of cationic channels (such as the cation channel subfamily M member 7 (TRPM7)), influencing the activation of intracellular signaling pathways (including the PIK3-dependent events) and enzymes and directly regulating transcription [107]. Notably, patients with X-linked immunodeficiency with magnesium defect, EBV infection, and neoplasia (XMEN) disease, which have a deficiency of intracellular magnesium, show uncontrolled EBV-related proliferation and immune dysregulation, increased risk of viral infections, and a high probability of developing EBV-related lymphoma [108,109].

Selenium acts mainly through the modulation of the function of selenoprotein, which includes enzymes involved in the antioxidant cellular defense, such as glutathione peroxidase [110]. Therefore, selenium has an important antioxidant role in leukocytes but also promotes Th1-mediated response (central against viral pathogens), as demonstrated by studies on mice supplemented with selenium, while the efficacy in enhancing antibody production is still controversial [111]. Moreover, supplementation with selenium allows the differentiation of macrophages into a tolerogenic subtype (M2 macrophages) and the generation of Tregs, thus limiting inflammatory damage [112]. Accordingly, studies on patients with selenium deficiency and animal models evidenced that this condition features reduced function of adaptive immunity accompanied by a higher degree of inflammation [110].

Coenzyme Q is a pivotal component of the mitochondrial electron transport chain, where it acts as an electron carrier but also has interesting antioxidant and anti-inflammatory properties. The antioxidant function of coenzyme Q depends on its capability to prevent lipid peroxidation and oxidative damage to nucleic acids and proteins and to regenerate vitamin E from α-tocopherol [113]. Additionally, it modulates different intracellular pathways through the regulation of the oxide reduction status of different enzymes (such as NADPH) [113,114]. Concerning the immune function, coenzyme Q directly interacts with transcriptional factors implicated in the immune response, including NF-kB and NRf2 [115], and inhibits the NLRP3 inflammasome complex [116], contrasting with the activation and enhancement of the inflammatory response. The positive influence on the PI3K molecular pathway, demonstrated by experimental models of acute lung injury, is important for the protection against oxidative stress and apoptosis [117], while the reduction of nitric oxide synthesis is responsible for the reduced inflammatory vascular response [113]. Finally, studies on animal models of GvHD have evidenced that supplementation with coenzyme Q causes an enhanced proliferation of Treg cells [118].

## 8. Conclusions

**Figure 3:**
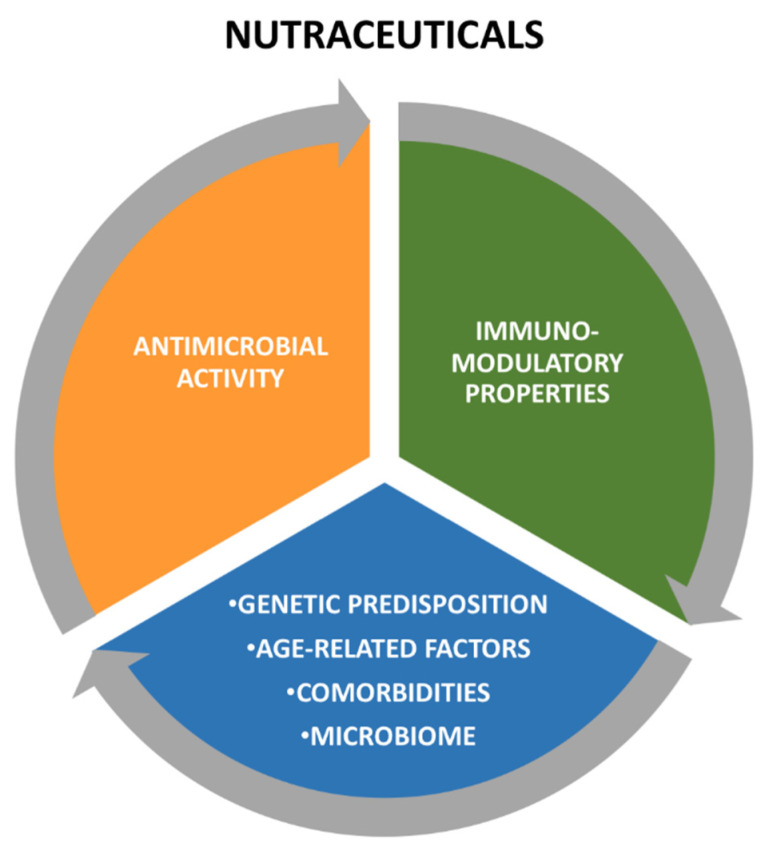
Interaction between individual substrate and nutraceuticals.

The actions of nutraceuticals on the immune system are extremely complex and not fully elucidated, and comprise a direct antimicrobial effect, the enhancement of the antiviral immune response, and immunomodulation that prevents tissue damage caused by the immune response itself. Although these actions seem to be conflicting, they contribute to maintaining immune homeostasis and optimizing the response to viral infections (Figure 3). Indeed, the different effects of the same molecule on the immune system could depend on its concentration, the cytokine environment, and the degree of systemic inflammation, thus highlighting the presence of a dynamic interaction between nutraceuticals and the immune system. The main antiviral actions and clinical evidence of the nutraceuticals addressed in this review are summarized in Table 1. However, many of these highlights derive from in vitro studies, since it is difficult to propose randomized clinical trials for molecules such as nutraceuticals. As a result, there is a lack of strong scientific evidence. As clinical data are still limited and not conclusive, further research is necessary to define the practical application of nutraceuticals in the prevention and treatment of viral infections.

## Figures and Tables

**Table 1:** Nutraceuticals and their relative antimicrobial properties.

Nutraceuticals	Effects on the Immune System	Main Targets
Vitamin D	- Modulation of the immune system - Stimulation of the synthesis of antimicrobial proteins (defensins and cathelicidins) - Increase of the chemotaxis of cells of innate immunity - Enhancement of epithelial barrier function - Modulation of the antioxidant system	SARS-CoV2 Respiratory Syncytial Virus
Vitamin A	- Regulation of NK cells, macrophages, and neutrophils - Proliferation, activity, and migration of APCs and cells of adaptive immunity - Modulation of the gut microbiome and stimulation of the synthesis of proteins involved in maintaining the integrity of the gastrointestinal barrier - Modulation of the function of the dendritic cells and their ability to present antigens - Maturation and development of tissue tropism of T and B lymphocytes - Stimulation of isotypic switch and IgA production	Respiratory viruses
Zinc	- Activation of NF-kB - Production of IFN and other antiviral proteins - Proliferation of macrophages, neutrophils, NK cells - Enhancement of NK cell lytic activity and phagocytosis - Promotion of antibody production and influencing of isotypic switch - Differentiation and proliferation of T cells	Influenza A virus Respiratory Syncytial Virus
Polyphenols	- Reduction of TLR expression - Reduction of NF-kB activation - Reduction of the release of pro-inflammatory cytokines after LPS stimulation, and the production of ROS	Influenza viruses (H1N1, H3N2, A/WS/33) Varicella Zoster Virus Cytomegalovirus
Lactoferrin	- Enhancement of T and NK lymphocyte activity - Activation of DCs and macrophage-enhancement of antigen-presenting function and secretion of cytokines - Intracellular inhibition of virus replication	Influenza A virus Parainfluenza virus Respiratory Syncytial Virus SARS-CoV2
Selenium, Magnesium, Coenzyme Q	- T-cell development, antibody production, and cytokine release and signaling - Antioxidant role - Promotion of Th1-mediated response - Limitation of inflammatory damage by stimulation of Tregs - Inhibition of the NLRP3 inflammasome	Respiratory viruses SARS-CoV2 Influenza A virus Epstein-Barr virus
